# Bioactive Evaluation of Ursane-Type Pentacyclic Triterpenoids: *β*-Boswellic Acid Interferes with the Glycosylation and Transport of Intercellular Adhesion Molecule-1 in Human Lung Adenocarcinoma A549 Cells

**DOI:** 10.3390/molecules27103073

**Published:** 2022-05-11

**Authors:** Kaori Nakano, Saki Sasaki, Takao Kataoka

**Affiliations:** Department of Applied Biology, Kyoto Institute of Technology, Matsugasaki, Sakyo-ku, Kyoto 606-8585, Japan; n.n.ka1025@gmail.com (K.N.); trumpetske@gmail.com (S.S.)

**Keywords:** pentacyclic triterpenoid, *β*-boswellic acid, uvaol, madecassic acid, AKBA, ICAM-1, glycosylation

## Abstract

Ursane-type pentacyclic triterpenoids exert various biological effects, including anticancer and anti-inflammatory activities. We previously reported that ursolic acid, corosolic acid, and asiatic acid interfered with the intracellular trafficking and glycosylation of intercellular adhesion molecule-1 (ICAM-1) in human lung adenocarcinoma A549 cells stimulated with the pro-inflammatory cytokine interleukin-1α. However, the structure–activity relationship of ursane-type pentacyclic triterpenoids remains unclear. In the present study, the biological activities of seven ursane-type pentacyclic triterpenoids (*β*-boswellic acid, uvaol, madecassic acid, 3-*O*-acetyl-11-keto-*β*-boswellic acid, ursolic acid, corosolic acid, and asiatic acid) were investigated. We revealed that the inhibitory activities of ursane-type pentacyclic triterpenoids on the cell surface expression and glycosylation of ICAM-1 and α-glucosidase activity were influenced by the number of hydroxy groups and/or the presence and position of a carboxyl group. We also showed that *β*-boswellic acid interfered with ICAM-1 glycosylation in a different manner from other ursane-type pentacyclic triterpenoids.

## 1. Introduction

Pro-inflammatory cytokines, such as interleukin-1 (IL-1) and tumor necrosis factor α (TNF-α), are produced at local sites in response to pathogen infections as well as physical and chemical stimuli, and trigger intracellular signaling pathways, one of which leads to the activation of the transcription factor nuclear factor κB (NF-κB) [1]. NF-κB induces the transcription of a number of genes that are essential for immune and inflammatory responses, including those encoding cytokines, chemokines, and cell adhesion molecules [2,3]. Intercellular adhesion molecule-1 (ICAM-1) is one of the major cell adhesion molecules primarily up-regulated by NF-κB in response to pro-inflammatory cytokines [4,5].

ICAM-1 belongs the immunoglobulin (Ig) superfamily and is expressed on the cell surface, in order to interact with β2-integrins, leukocyte function-associated antigen 1 (CD11a/CD18), and macrophage antigen 1 (CD11b/CD18) via its Ig domains [6]. In vascular endothelial cells, ICAM-1 is expressed at basal levels and is up-regulated by pro-inflammatory cytokines [7]. In addition to other adhesion molecules, ICAM-1 plays an essential role in the recruitment and the transendothelial migration of circulating leukocytes [8,9]. Human ICAM-1 possesses eight *N*-glycosylation sites, and is heavily glycosylated with complex-type *N*-glycans [6,7]. *N*-glycosylation is essential for the intracellular trafficking of ICAM-1 because tunicamycin has been shown to prevent the cell-surface expression of non-*N*-glycosylated ICAM-1 [10,11]. Previous studies demonstrated that high mannose-type ICAM-1 enhanced adhesion to monocytes [12,13]. Therefore, ICAM-1 helps to regulate cell–cell interactions by its cell-surface level and glycan forms, which in turn influence inflammatory and immune responses.

**Figure 1:**
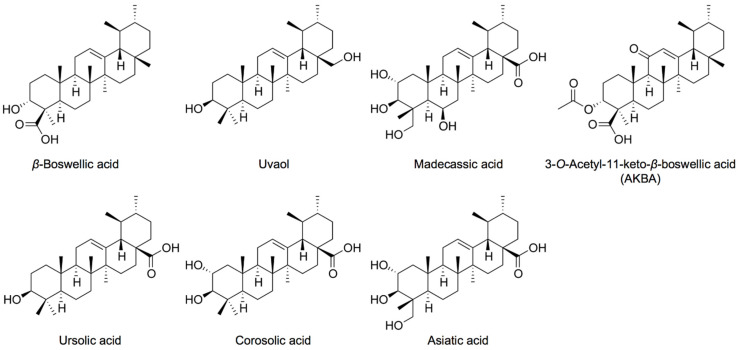
Structures of ursane-type pentacyclic triterpenoids.

Hydroxy pentacyclic triterpene acids (HPTAs) and their derivatives are secondary metabolites that are often found in plants, and have been shown to exert diverse biological effects, such as anti-inflammatory and anti-tumor activities [14,15,16]. We previously revealed that six HPTAs (Figure S1) interfered with different steps in the *N*-glycosylation and intracellular transport of glycoproteins [11,17,18]. Ursolic acid (a ursane-type HPTA) inhibited the transport of ICAM-1 from the endoplasmic reticulum (ER) to the Golgi apparatus [11], while its structural isomers, oleanolic acid (an oleanane-type HPTA) and betulinic acid (a lupane-type HPTA), did not suppress the cell-surface expression of ICAM-1 [17]. Corosolic acid (a ursane-type HPTA) and, to a lesser extent, maslinic acid (an oleanane-type HPTA) inhibited the cell-surface expression of ICAM-1, whereas asiatic acid (a ursane-type HPTA) delayed the transport of ICAM-1 to the cell surface [18]. These findings suggest that ursane-type HPTAs interfere with the *N*-glycosylation and intracellular transport of glycoproteins with various efficacies and different modes of action. However, the structure–activity relationship of ursane-type HPTAs in these processes remains unclear. In addition to the three HPTAs previously examined (i.e., ursolic acid, corosolic acid, and asiatic acid), we investigated the biological activities of three ursane-type HPTAs (i.e., *β*-boswellic acid, 3-*O*-acetyl-11-keto-*β*-boswellic acid (AKBA), and madecassic acid) and uvaol in order to elucidate the structure–activity relationship of ursane-type pentacyclic triterpenoids in more detail (Figure 1). In the present study, we also revealed that *β*-boswellic acid interfered with the glycosylation and transport of ICAM-1 in a different manner from other ursane-type pentacyclic triterpenoids.

## 2. Results and Discussion

### 2.1. Bioactive Evaluation of Ursane-Type Pentacyclic Triterpenoids on the Viability of A549 Cells

**Figure 2:**
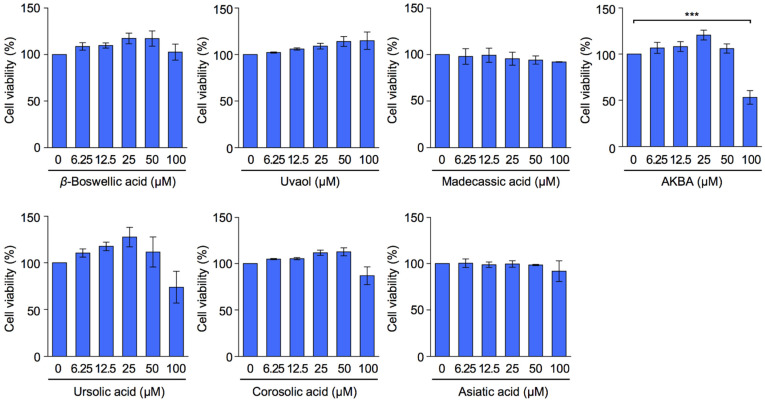
Effects of ursane-type pentacyclic triterpenoids on cell viability. A549 cells were incubated with serial dilutions of triterpenoids for 7 h at the indicated concentrations. Cell viability was evaluated by the MTT assay. Cell viability (%) is shown as the mean ± S.E. of three independent experiments. *** *p* < 0.001.

We previously showed that three ursane-type HPTAs (i.e., ursolic acid, corosolic acid, and asiatic acid) exerted different effects on the expression and glycosylation of ICAM-1 in human lung adenocarcinoma A549 cells stimulated with IL-1α [11,18]. IL-1α and IL-1β have been shown to stimulate the same IL-1 receptor and trigger the same signaling pathway [19,20]. In the present study, we also used four ursane-type pentacyclic triterpenoids (i.e., *β*-boswellic acid, AKBA, madecassic acid, and uvaol) to investigate the structure–activity relationship. A549 cells were incubated with seven triterpenoids for 7 h. Cell viability was evaluated using the 3-(4,5-dimethylthizol-2-yl)-2,5-diphenyltetrazolium bromide (MTT) assay. The effects of *β*-boswellic acid, uvaol, madecassic acid, and asiatic acid on cell viability were negligible at concentrations up to 100 µM (Figure 2). Furthermore, ursolic acid and corosolic acid did not markedly affect cell viability at 100 µM. In contrast, AKBA decreased cell viability by ~50% at 100 µM up to 7 h. These results showed that AKBA exerted the strongest cytotoxic effect on A549 cells. This is consistent with previous findings indicating that AKBA reduced the viability of A549 cells at lower concentrations when they were incubated for longer durations (24 to 72 h) [21,22].

### 2.2. Bioactive Evaluation of Ursane-Type Pentacyclic Triterpenoids on Cell-Surface ICAM-1 Expression

**Figure 3:**
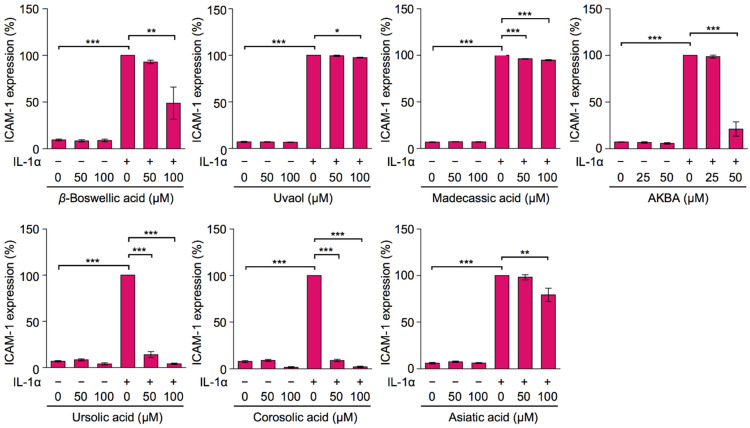
Effects of ursane-type pentacyclic triterpenoids on cell-surface ICAM-1 expression. A549 cells were preincubated with serial dilutions of triterpenoids for 1 h, and then incubated with (+) or without (−) IL-1α (0.25 ng/mL) for 6 h in the presence of triterpenoids at the indicated concentrations. ICAM-1 expression was evaluated by cell-ELISA. ICAM-1 expression (%) is shown as the mean ± S.E. of three independent experiments. * *p* < 0.05, ** *p* < 0.01, and *** *p* < 0.001.

To investigate the structure–activity relationship of cell-surface ICAM-1 expression, A549 cells were preincubated with triterpenoids for 1 h followed by IL-1α for 6 h, and a cell-enzyme-linked immunosorbent assay (ELISA) was then performed to quantitate the cell-surface expression of ICAM-1. Consistent with our previous findings [11,18], the cell-surface expression of ICAM-1 was inhibited by ursolic acid and corosolic acid at concentrations higher than 50 µM, and was slightly suppressed by asiatic acid at 100 µM (Figure 3). Asiatic acid at 100 µM up-regulated the expression of ICAM-1 protein, increased the co-localization of ICAM-1 with the ER marker calnexin, and delayed the expression of cell-surface ICAM-1 [18]. These biological activities of asiatic acid may explain the slight suppression of cell-surface ICAM-1 expression by asiatic acid at 100 µM. Madecassic acid and uvaol did not markedly decrease the cell-surface expression of ICAM-1 (Figure 3). *β*-Boswellic acid at 100 µM reduced the cell-surface expression of ICAM-1 by ~50%. AKBA at 50 µM inhibited the cell-surface expression of ICAM-1 by ~80%. These results showed that ursolic acid and corosolic acid, which possess one and two hydroxy groups, exhibited the strongest inhibitory activities on the cell-surface expression of ICAM-1 with lower cytotoxicity than AKBA. Regarding the structure–activity relationship of ursane-type triterpenoids on the cell-surface expression of ICAM-1, the present results revealed the following: (1) inhibitory activity required a carboxyl group (based on a comparison between ursolic acid and uvaol); (2) inhibitory activity was weakened by the presence of additional hydroxy groups (based on a comparison of three and four hydroxy groups in asiatic acid and madecassic acid with ursolic acid and corosolic acid); and (3) inhibitory activity was influenced by the position of the carboxyl group (based on a comparison between ursolic acid and *β*-boswellic acid).

### 2.3. Bioactive Evaluation of Ursane-Type Pentacyclic Triterpenoids on ICAM-1 N-Glycosylation

**Figure 4:**
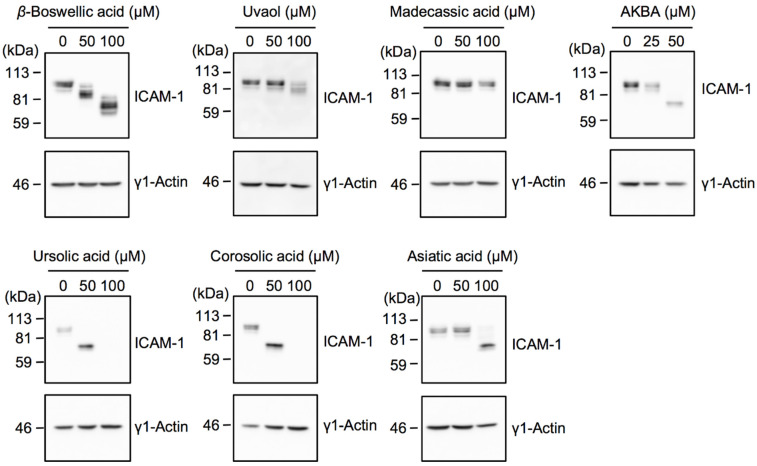
Effects of ursane-type pentacyclic triterpenoids on the glycosylation and expression of ICAM-1. A549 cells were preincubated with triterpenoids for 1 h and then incubated with IL-1α (0.25 ng/mL) for 6 h in the presence of triterpenoids at the indicated concentrations. The ICAM-1 protein was evaluated by Western blotting. Blots are representative of at least three independent experiments.

Human ICAM-1 possesses eight *N*-glycosylation sites [6]. In A549 cells, fully glycosylated ICAM-1 was observed as a heterogenous band migrating at more than 80 kDa, while unglycosylated ICAM-1 caused by a tunicamycin treatment or digestion by a peptide: *N*-glycosidase F (PNGase F) was detected as a band migrating at 50–55 kDa [11]. To investigate the structure–activity relationship of ICAM-1 glycosylation, the molecular size of the ICAM-1 protein was evaluated by Western blotting. Consistent with our previous findings [11,18], ursolic acid, corosolic acid, and asiatic acid decreased the molecular size of ICAM-1 to between 59 and 81 kDa (Figure 4). Ursolic acid and corosolic acid at 100 µM down-regulated ICAM-1 protein expression. In contrast, madecassic acid at 100 µM did not affect the molecular size of ICAM-1. *β*-Boswellic acid at 50 and 100 µM induced step-by-step decreases in the molecular size of ICAM-1. Uvaol at 100 µM only slightly affected the molecular size of ICAM-1. AKBA at 50 µM decreased the molecular size of ICAM-1 to between 59 and 81 kDa. Since madecassic acid and uvaol did not exert marked effects on the molecular size of ICAM-1 among seven triterpenoids, the carboxyl group of ursane-type triterpenoids appeared to be essential for interfering with ICAM-1 glycosylation, and this biological activity was also attenuated by increases in hydroxy groups.

### 2.4. Bioactive Evaluation of Ursane-Type Pentacyclic Triterpenoids on α-Glucosidase Activity

**Figure 5:**
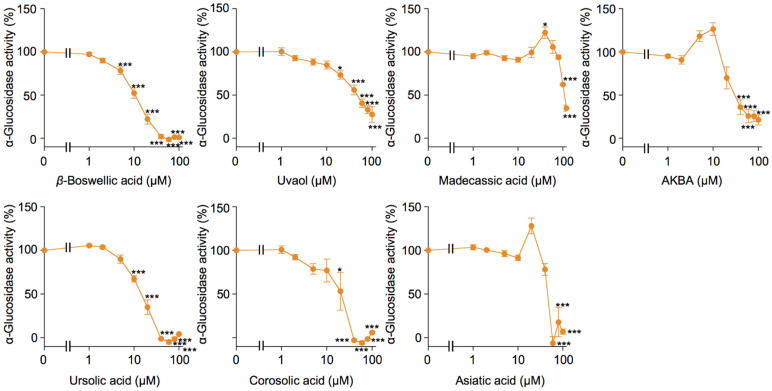
Effects of ursane-type pentacyclic triterpenoids on α-glucosidase activity. Yeast α-glucosidase was preincubated with triterpenoids for 30 min, and then incubated with *p*-nitrophenyl-α-D-glucopyranoside for 15 min in the presence of triterpenoids. α-Glucosidase activity (%) was shown as the mean ± S.E. of three independent experiments. * *p* < 0.05, and *** *p* < 0.001.

In *N*-glycan modifications, three glucose residues are removed by ER α-glucosidases I and II from the Glucose_3_Mannose_9_GlcNAc_2_ glycans after their transfer to proteins in the ER, which was prevented by castanospermine [23]. While betulinic acid and oleanolic acid did not prevent the cell-surface expression of ICAM-1, they interfered with its *N*-glycan modifications, similar to castanospermine in A549 cells [17]. Yeast α-glucosidase activity was inhibited by betulinic acid and oleanolic acid at 50% inhibitory concentration (IC_50_) values of 10.5 and 54.3 µM [17]. Therefore, betulinic acid and oleanolic acid appear to prevent glucose trimming by α-glucosidases in the ER. We previously reported that the IC_50_ value of ursolic acid on yeast α-glucosidase was 20.1 µM [17]. We also showed that the IC_50_ values of corosolic acid and asiatic acid were 8.5 and 24.4 µM [18]. To elucidate the structure–activity relationship in more detail, the inhibitory activities of seven triterpenoids on α-glucosidase were measured and directly compared. Ursolic acid, corosolic acid, and asiatic acid exerted inhibitory activities at IC_50_ values of 20.5 ± 4.9, 18.2 ± 4.7, and 45.9 ± 1.4 µM (Figure 5), indicating that ursolic acid and corosolic acid inhibited α-glucosidase with similar efficacies. *β*-Boswellic acid and AKBA inhibited α-glucosidase activity at IC_50_ values of 11.1 ± 1.4 and 30.1 ± 6.0 µM. The IC_50_ values of madecassic acid and uvaol were 108.9 ± 1.7 and 47.9 ± 7.0 µM. These results showed that *β*-boswellic acid exhibited a stronger inhibitory activity against α-glucosidase compared to ursolic acid and corosolic acid. *β*-Boswellic acid has been reported to inhibit α-glucosidase at the IC_50_ value of 49.726 ± 1.224 µM [24], while it has been also shown that *β*-boswellic acid is not active for α-glucosidase up to 1 mM [25]. Our present results also revealed that the carboxyl group of ursane-type pentacyclic triterpenoids is important for the inhibitory activity of α-glucosidase, which was attenuated by increases in hydroxy groups. This is consistent with previous findings for the structure–activity relationship of α-glucosidase: corosolic acid exhibited a stronger inhibitory activity than asiatic acid [26,27,28], while ursolic acid more strongly inhibited α-glucosidase than uvaol [29,30].

### 2.5. β-Boswellic Acid Altered ICAM-1 Glycosylation in a Different Manner from Other Ursane-Type Pentacyclic Triterpenoids

**Figure 6:**
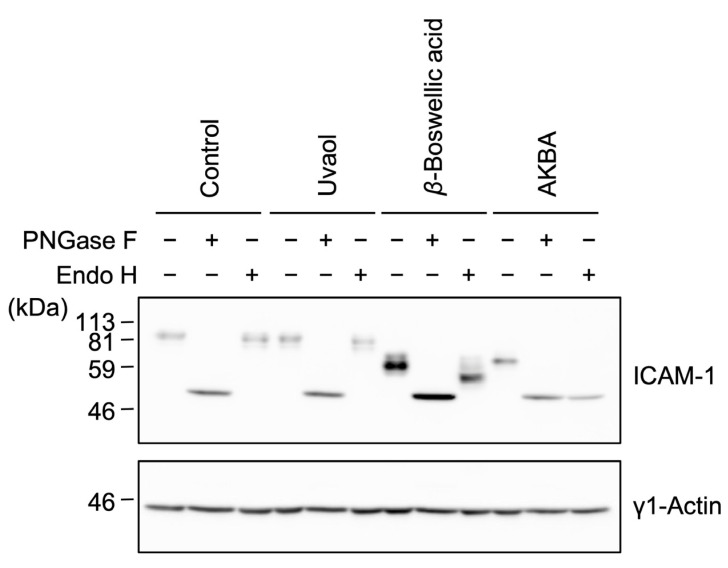
Effects of ursane-type pentacyclic triterpenoids on the Endo H sensitivity of ICAM-1 *N*-glycans. A549 cells were preincubated with triterpenoids for 1 h, and then incubated with IL-1α (0.25 ng/mL) for 6 h in the presence of uvaol (100 µM), *β*-boswellic acid (100 µM) or AKBA (50 µM). Cell lysates were treated with (+) or without (−) PNGase F or Endo H and then analyzed by Western blotting. Blots are representative of three independent experiments.

Figure 3 and Figure 4 suggested that *β*-boswellic acid interfered with ICAM-1 glycosylation and/or intracellular transport in a different manner from ursolic acid, corosolic acid, and asiatic acid. High-mannose-type glycans are attached to proteins in the ER and processed to complex-type and hybrid-type glycans by the removal and addition of sugars in the ER and Golgi apparatus. PNGase F digests high mannose-type, hybrid-type, and complex-type glycans, while Endoglycosidase H (Endo H) digests high mannose-type and some hybrid-type glycans. We previously showed that ICAM-1 proteins in ursolic-acid-, corosolic-acid-, or asiatic-acid-treated cells were digested by both Endo H and PNGase F to a similar size [18], indicating that Endo-H-sensitive glycans are mainly attached to ICAM-1 upon exposure to ursolic acid, corosolic acid, and asiatic acid. In control cells, the ICAM-1 protein was not cleaved by Endo H, indicating that Endo-H-resistant glycans were mainly attached to ICAM-1 (Figure 6). In uvaol-treated cells, the digestion of the ICAM-1 protein by Endo H was negligible. In AKBA-treated cells, the ICAM-1 protein was digested by both Endo H and PNGase F to a similar size, indicating that Endo-H-sensitive glycans were mainly attached to ICAM-1. The effect of AKBA on ICAM-1 *N*-glycosylation seemed to be similar to that of ursolic acid, corosolic acid, and asiatic acid [11,18]. In *β*-boswellic-acid-treated cells, Endo H digested the ICAM-1 protein to major bands, which were larger than those digested by PNGase F (Figure 6). These results show that Endo-H-sensitive glycans and Endo-H-resistant glycans both attached to the ICAM-1 protein in *β*-boswellic-acid-treated cells and that the effects of *β*-boswellic acid on ICAM-1 *N*-glycosylation differed from those of ursolic acid, corosolic acid, and asiatic acid [11,18].

### 2.6. β-Boswellic Acid Induced the Localization of ICAM-1 at the Perinuclear Region

**Figure 7:**
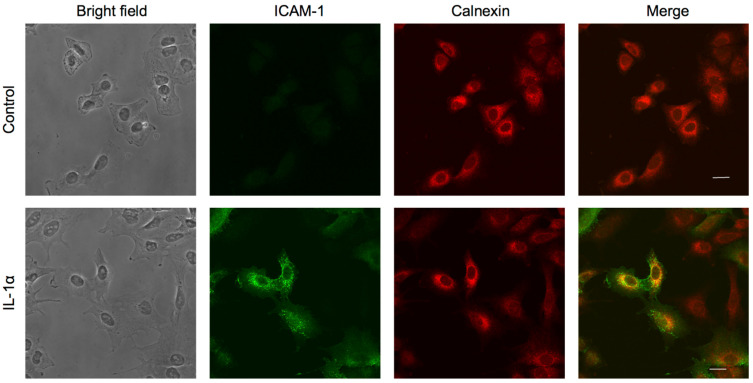

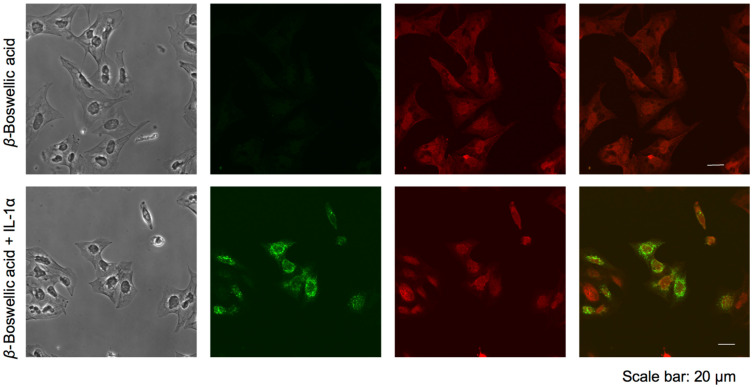
Effects of *β*-boswellic acid on the subcellular localization of ICAM-1 and calnexin. A549 cells were preincubated with (+) or without (−) *β*-boswellic acid for 1 h and then incubated with (+) or without (−) IL-1α (0.25 ng/mL) for 6 h in the presence or absence of *β*-boswellic acid (100 µM). Cells were stained with the mouse anti-ICAM-1 antibody and Alexa Fluor^®^ 488-labeled anti-mouse IgG antibody or rabbit anti-calnexin and Alexa Fluor^®^ 594-labled anti-rabbit IgG antibody. Due to different acquirement conditions for clearer images, the subcellular localization of ICAM-1 and calnexin was observed, while fluorescent intensities between images were not directly compared. Data are representative of three independent experiments. Scale bars: 20 µm.

To investigate the subcellular localization of ICAM-1, A549 cells were preincubated with *β*-boswellic acid for 1 h, stimulated with IL-1α for 6 h, and then immunostained with antibodies reactive to ICAM-1, with calnexin as the ER marker. This IL-1α stimulation induced the expression of ICAM-1, which was detected as small puncta over the cytoplasm and at the cell periphery (Figure 7). Calnexin mainly localized to the perinuclear region, a part of which co-localized with ICAM-1. These results showed that ICAM-1 was expressed in the ER, the cell membrane, and other intracellular regions. In contrast, *β*-boswellic acid caused ICAM-1 puncta mainly at the perinuclear region (Figure 7). The perinuclear localization of calnexin was not clearly observed in *β*-boswellic-acid-treated cells. These results suggested that *β*-boswellic acid interfered with the intracellular transport of ICAM-1 mainly at the ER, which may be ascribed to a partial reduction in cell-surface ICAM-1 expression.

### 2.7. β-Boswellic Acid Interferes with ICAM-1 Glycosylation and Intracellular Transport in a Different Manner from Other Ursane-Type Pentacyclic Triterpenoids

We previously showed that ursolic acid inhibited the transport of ICAM-1 from the ER to the Golgi apparatus and induced the accumulation of Endo-H-sensitive ICAM-1 in the ER [11]. Asiatic acid delayed the transport of ICAM-1 from the ER, and allowed the expression of Endo-H-sensitive ICAM-1 at the cell surface [18]. In the present study, we showed that *β*-boswellic acid induced the expression of ICAM-1 possessing Endo-H-sensitive and Endo-H-resistant glycans, and this was accompanied by a partial reduction in the cell-surface expression of ICAM-1. These results revealed that the mechanisms of action of *β*-boswellic acid differed from those of ursolic acid and asiatic acid. We also previously demonstrated that ICAM-1 accumulated at the perinuclear regions, and co-localized with calnexin in ursolic-acid-treated cells [11]. In asiatic-acid-treated cells, ICAM-1 co-localized with calnexin in the perinuclear region [18]. However, calnexin was not clearly observed at the perinuclear region in *β*-boswellic-acid-treated cells. This is consistent with the notion that *β*-boswellic acid impairs the morphology of the ER and/or the expression of ER-resident proteins in a different manner from ursolic acid or asiatic acid. Boswellic acid and its analogues have been shown to inhibit many molecular targets, such as transcription factors, protein kinases, receptors, and enzymes, for its anti-inflammatory and anticancer activities [31,32,33]. Nevertheless, the molecular targets of *β*-boswellic acid for glycoprotein transport and *N*-glycosylation remain unclear. Therefore, the effects of *β*-boswellic acid on ER processes and components warrant further study.

## 3. Materials and Methods

### 3.1. Cell Culture

Human lung adenocarcinoma A549 cells (JCRB0076) were obtained from the National Institutes of Biomedical Innovation, Health and Nutrition JCRB Cell Bank (Osaka, Japan). A549 cells were cultured with RPMI 1640 medium (Thermo Fisher Scientific, Gland Island, NY, USA) supplemented with heat-inactivated fetal calf serum (Sigma-Aldrich, St. Louis, MO, USA) and a penicillin-streptomycin antibiotic mixture (Nacalai Tesque, Kyoto, Japan).

### 3.2. Reagents

Asiatic acid (Tokyo Chemical Industry Co., Ltd., Tokyo, Japan), *β*-boswellic acid (Cayman Chemical Company, Ann Arbor, MI, USA), corosolic acid (Cayman Chemical Company), madecassic acid (Cayman Chemical Company), AKBA (Alexis Corporation, Lausen, Switzerland), ursolic acid (Sigma-Aldrich), and uvaol (Sigma-Aldrich) were used in this study. Recombinant human IL-1α was provided by Dainippon Pharmaceutical Co., Ltd. (Osaka, Japan).

### 3.3. Antibodies

Primary antibodies for β-actin (AC-15; Sigma-Aldrich), γ1-actin (2F3; FUJIFILM Wako Pure Chemical Corporation, Osaka, Japan), calnexin (ab92573; Abcam, Cambridge, UK), ICAM-1 (15.2; Leinco Technologies, Inc., St. Louis, MO, USA), ICAM-1 (28; BD Biosciences, San Diego, CA, USA), peroxidase-conjugated anti-mouse IgG(H+L) antibody (Jackson ImmunoResearch Laboratories, West Grove, PA, USA), Alexa^®^ 488-conjugated anti-mouse IgG antibody (Thermo Fisher Scientific), and Alexa^®^ 594-conjugated anti-rabbit IgG antibody (Thermo Fisher Scientific) as secondary antibodies were used in this study.

### 3.4. Cell Viability Assay

A549 cells (100 µL/well) were incubated for the last 2 h after the addition of 10 µL of MTT-phosphate-buffered saline (PBS) (0.5 mg/mL). To solubilize MTT formazan, A549 cells were incubated overnight after the addition of 100 µL of 10% SDS. Absorbance at 570 nm was measured using the iMark^TM^ microplate reader (Bio-Rad Laboratories, Hercules, CA, USA).

### 3.5. Cell-ELISA

A549 cells were washed twice with PBS, fixed with 1% paraformaldehyde–PBS for 15 min, washed twice with PBS, and incubated with 1% bovine serum albumin (BSA)–PBS overnight. A549 cells were incubated with anti-ICAM-1 antibody (15.2) in BSA–PBS for 1 h, washed three times with 0.02% Tween 20–PBS, and then incubated with peroxidase-conjugated anti-mouse IgG(H+L) antibody in BSA–PBS for 1 h. A549 cells were washed three times with 0.02% Tween 20–PBS and then incubated with substrate solution (0.2 M sodium citrate buffer (pH 5.3), 0.1% *o*-phenylenediamine hydrochloride, and 0.02% H_2_O_2_) until color development. Absorbance at 450 nm was measured by the iMark^TM^ microplate reader.

### 3.6. Western Blotting

A549 cells were washed with PBS and lyzed by Triton X-100 lysis buffer (50 mM Tris-HCl (pH 7.5), 1% Triton X-100, 2 mM dithiothreitol, and 2 mM sodium vanadate) containing cOmplete^TM^ (Sigma-Aldrich) on ice for 15 min, followed by centrifugation (15,300× *g*, 5 min). Cell lysates were collected as supernatants. Cell lysates (30 µg protein) were separated by SDS-PAGE and transferred to ClearTrans^®^ 0.22-µm nitrocellulose membranes (FUJIFILM Wako Pure Chemical Corporation). Membranes were incubated with 5% skim milk in 0.5% Tween 20–PBS (PBS-T) overnight at 4 °C. Membranes were then incubated with primary antibodies and peroxidase-conjugated secondary antibodies. Protein bands were visualized by Amersham Imager 680 (GE Healthcare Japan, Tokyo, Japan) and analyzed by ImageQuant TL software (GE Healthcare Japan).

### 3.7. α-Glucosidase Assay

α-Glucosidase from yeast (0.005U) (FUJIFILM Wako Pure Chemical Corporation) was preincubated with triterpenoids at room temperature for 30 min and then incubated with 50 mM sodium phosphate buffer (pH 6.8) containing *p*-nitrophenyl-α-D-glucopyranoside (0.5 mM) (FUJIFILM Wako Pure Chemical Corporation) at 37 °C for 15 min. Absorbance at 405 nm was measured using the iMark^TM^ microplate reader.

### 3.8. Glycosidase Digestion

Cell lysates (20 µg of protein) were denatured and then incubated with PNGase F (New England BioLabs, Ipswich, MA, USA) or Endo H (New England Biolabs) according to the manufacturers’ protocols. Cell lysates were separated by SDS-PAGE and analyzed by Western blotting.

### 3.9. Immunostaining

A549 cells were grown on coverslips coated with Cellmatrix type I-C (Nitta Gelatin, Osaka, Japan). A549 cells were fixed with 4% PFA–PBS for 15 min, washed serially with PBS, 25 mM glycine-PBS, and 0.3% Triton X-100–PBS (PBS-T), followed by blocking with 5% normal goat serum–PBS-T. Cells were incubated with the mouse anti-ICAM-1 antibody and rabbit anti-calnexin antibody, followed by the Alexa^®^ 488-conjugated anti-mouse IgG antibody (Thermo Fisher Scientific) and Alexa^®^ 594-conjugated anti-rabbit IgG antibody (Thermo Fisher Scientific). Images were acquired using a confocal laser scanning microscope system FV10i (Olympus, Tokyo, Japan).

### 3.10. Statistical Analysis

A one-way ANOVA and Tukey’s post hoc test were performed for multiple comparisons using KaleidaGraph 4.5 software (Hulinks, Tokyo, Japan).

## 4. Conclusions

Ursane-type HPTAs interfered with the cell-surface expression and glycosylation of ICAM-1. Due to the essential role of ICAM-1 in inflammatory responses, the development of ursane-type HPTAs as anti-inflammatory agents was expected. Ursolic acid and corosolic acid were the strongest inhibitors of the cell-surface expression of ICAM-1. Asiatic acid delayed the cell-surface expression of ICAM-1, while madecassic acid did not affect the cell-surface expression or glycosylation of ICAM-1. In conclusion, this structure–activity relationship study revealed that the number of hydroxy groups and the presence and/or position of a carboxyl group attached to ursane-type HPTAs influenced their biological effects on the transport and glycosylation of ICAM-1. The preparation of ursane-type pentacyclic derivatives with protected hydroxy groups (for example, acetate) and their bioactive evaluation is important for confirming our proposed results. We also demonstrated that *β*-boswellic acid interfered with the transport and glycosylation of ICAM-1 in a different manner from other ursane-type pentacyclic triterpenoids. Further studies to elucidate the mechanisms of action of HPTAs on the glycosylation and intracellular glycoprotein transport are needed for the development of more effective anti-inflammatory agents.

## Data Availability

The data presented in the present study are available upon request from the corresponding author.

## Supplementary Materials

The following supporting information can be downloaded at: https://www.mdpi.com/article/10.3390/molecules27103073/s1, Figure S1: Structures of HPTAs.
