# Correction: Nakano et al. Bioactive Evaluation of Ursane-Type Pentacyclic Triterpenoids: *β*-Boswellic Acid Interferes with the Glycosylation and Transport of Intercellular Adhesion Molecule-1 in Human Lung Adenocarcinoma A549 Cells. *Molecules* 2022, *27*, 3073

**DOI:** 10.3390/molecules30132724

**Published:** 2025-06-25

**Authors:** Kaori Nakano, Saki Sasaki, Takao Kataoka

**Affiliations:** Department of Applied Biology, Kyoto Institute of Technology, Matsugasaki, Sakyo-ku, Kyoto 606-8585, Japan

In the original publication [1], there was a mistake in Figure 6 as published. The 1st, 4th, 7th and 10th lanes should be marked with PNGase F (−) and Endo H (−). The corrected Figure 6 appears below.

The authors state that the scientific conclusions are unaffected. This correction was approved by the Academic Editors. The original publication has also been updated.

## Figures and Tables

**Figure 6:**
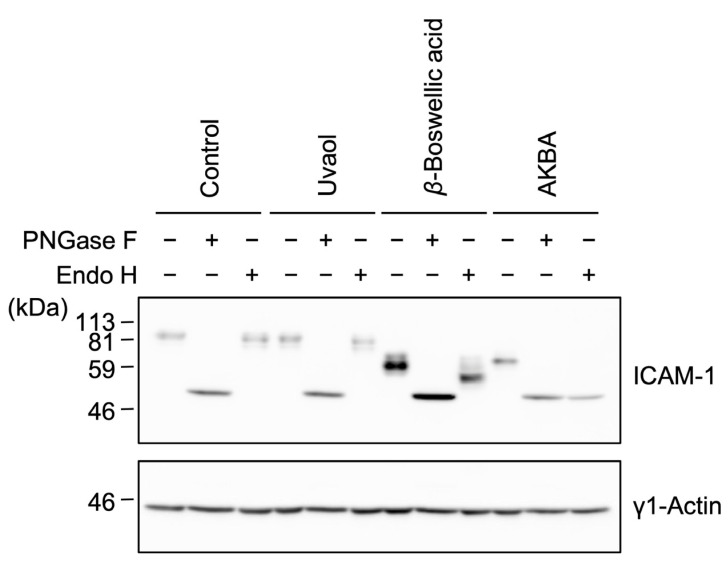
Effects of ursane-type pentacyclic triterpenoids on the Endo H sensitivity of ICAM-1 *N*-glycans. A549 cells were preincubated with triterpenoids for 1 h, and then incubated with IL-1α (0.25 ng/mL) for 6 h in the presence of uvaol (100 µM), *β*-boswellic acid (100 µM) or AKBA (50 µM). Cell lysates were treated with (+) or without (−) PNGase F or Endo H and then analyzed by Western blotting. Blots are representative of three independent experiments.
