# Supplementary figures and images for: Bioactive Evaluation of Ursane-Type Pentacyclic Triterpenoids: β-Boswellic Acid Interferes with the Glycosylation and Transport of Intercellular Adhesion Molecule-1 in Human Lung Adenocarcinoma A549 Cells
Source: Molecules. 2022 May 11;27(10):3073. doi: 10.3390/molecules27103073 (PMC9147781; doi:10.3390/molecules27103073)

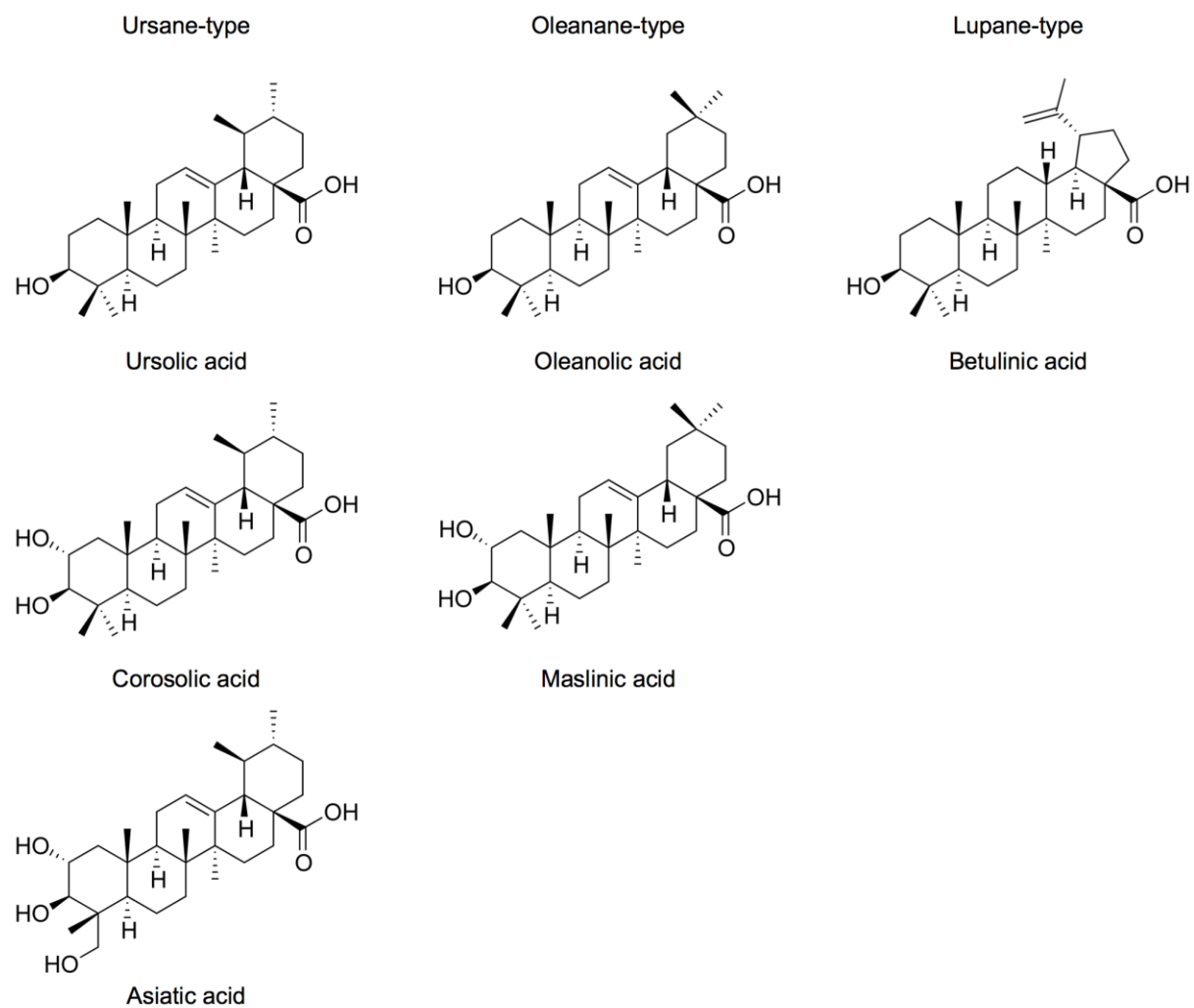

**Figure S1.** Structures of HPTAs.

Supplement: Supplementary file 1 [file molecules-27-03073-s001.zip › molecules-1726732-supplementary.pdf]
